# Exosomal proteins: new targets for early diagnosis and treatment of cancer

**DOI:** 10.3389/fimmu.2025.1613494

**Published:** 2025-06-19

**Authors:** Haibin Shen, Meijin Liu, Wentai Yang, Dewang Xiao, Zongbo Peng, Dingyu Rao, Defa Huang

**Affiliations:** ^1^ Laboratory Medicine, First Affiliated Hospital of Gannan Medical University, Ganzhou, China; ^2^ Laboratory Medicine, People’s Hospital of Ganzhou Economic Development Zone, Ganzhou, China; ^3^ Department of Gastroenterology, First Affiliated Hospital of Gannan Medical University, Ganzhou, China; ^4^ The First School of Clinical Medicine, Gannan Medical University, Ganzhou, China; ^5^ Department of Cardiothoracic Surgery, First Affiliated Hospital of Gannan Medical University, Ganzhou, China

**Keywords:** exosomes, exosomal proteins, biomarkers, cancer diagnosis, treatment

## Abstract

Exosomes are nanoscale, double-membraned vesicles released by a variety of living cells. A wide variety of cargoes, including proteins, DNA and RNA, are transported by exosomes to target cells, thereby transmitting biological signals. In addition to being an essential component of the exosomal cargo, exosomal proteins are a reflection of the physiological state of the originating cell, and play an essential part in intercellular communication in numerous diseases, including cancer. The present review provides a summary of the novel uses of exosomal proteins in cancer diagnosis and prognosis, and highlights the distinct qualities that exosomal proteins possess, when compared with typical serological measurements.

## 1 Introduction

Cancer is a leading cause of death worldwide. At present, tissue biopsy is considered the gold standard method for the clinical diagnosis of cancer. However, tissue collection may be complex, due to the close proximity of some tumors to major arteries in the body. In addition, obtaining tumor tissues from a specific location may not accurately represent the overall health of the patient (1). By contrast, liquid biopsies, which include biopsies of circulating tumor cells (CTCs), free DNA (cfDNA) and exosomes, provide further information regarding the status of the tumor, and do not require invasive procedures (2). This information may be obtained through frequent non-invasive sampling, which aids in the diagnosis of cancer and monitoring the pathophysiologic condition of the patient (3, 4).

Exosomes are biologically active extracellular vesicles that range from 50–200 nanometers in size, and are produced by live cells (5). Exosomes are located in the majority of bodily fluids, including urine, blood, saliva, ascites, breast milk, amniotic fluid and cerebrospinal fluid (6). Exosomes are responsible for transporting a variety of substances (7, 8), including proteins, nucleic acids, lipids, enzymes and metabolites, and these are regulated by the physiology of the original cell (9). Several mechanisms, including direct fusion with cell membranes, endocytosis routes and ligand-receptor interactions, are involved in the process of intercellular communication (10–12). Notably, exosomes may play a role in intercellular communication.

As tumor cells are primarily responsible for the production of tumor-derived exosomes that contain chemicals which reflect the properties of the parental tumor cells (13), they may exhibit potential as diagnostic markers for tumors (14). Importantly, liquid biopsy of exosomes may be more useful than CTC or circulating tumor DNA (ctDNA), as there is a large volume of exosomes in bodily fluids (up to 10^11^ exosomes per ml in blood), making them easier to detect. In addition, ~10% of exosomes detected in the bodily fluids of individuals who have advanced malignancies originate from tumor cells (15). At present, research is focused on exosomal RNAs, including microRNAs, circular RNAs (circRNA) and long non-coding RNAs (16–18). Exosomal proteins are either encased inside an inner lumen or embedded on the surface of the exosome. This allows for the categorization of exosome subtypes based on surface biomarkers, without causing any disruption to the structure of the exosomes. Compared with alternate exosomal cargoes, exosomal proteins exhibit numerous advantages, including i) a long half-life and stability inside the exosomes in which they are found (19), and ii) a relatively straightforward isolation procedure, due to identification of exosomal surface proteins in smaller sample sizes (20–22). Through the use of proteomics, protein coverage and sensitivity have been significantly enhanced, which has led to an increase in the breadth of exosomal proteomics data surrounding cancer causation, function and disease association (23). Further investigations are required to determine the specific process of carcinogenesis and the advancement of cancer. The present study aimed to provide a summary of the diagnostic and prognostic functions that exosomal proteins play in a variety of malignancies, and demonstrate the potential uses of exosomal proteins in clinical cancer treatment.

## 2 Biogenesis of exosomes

**Figure 1:**
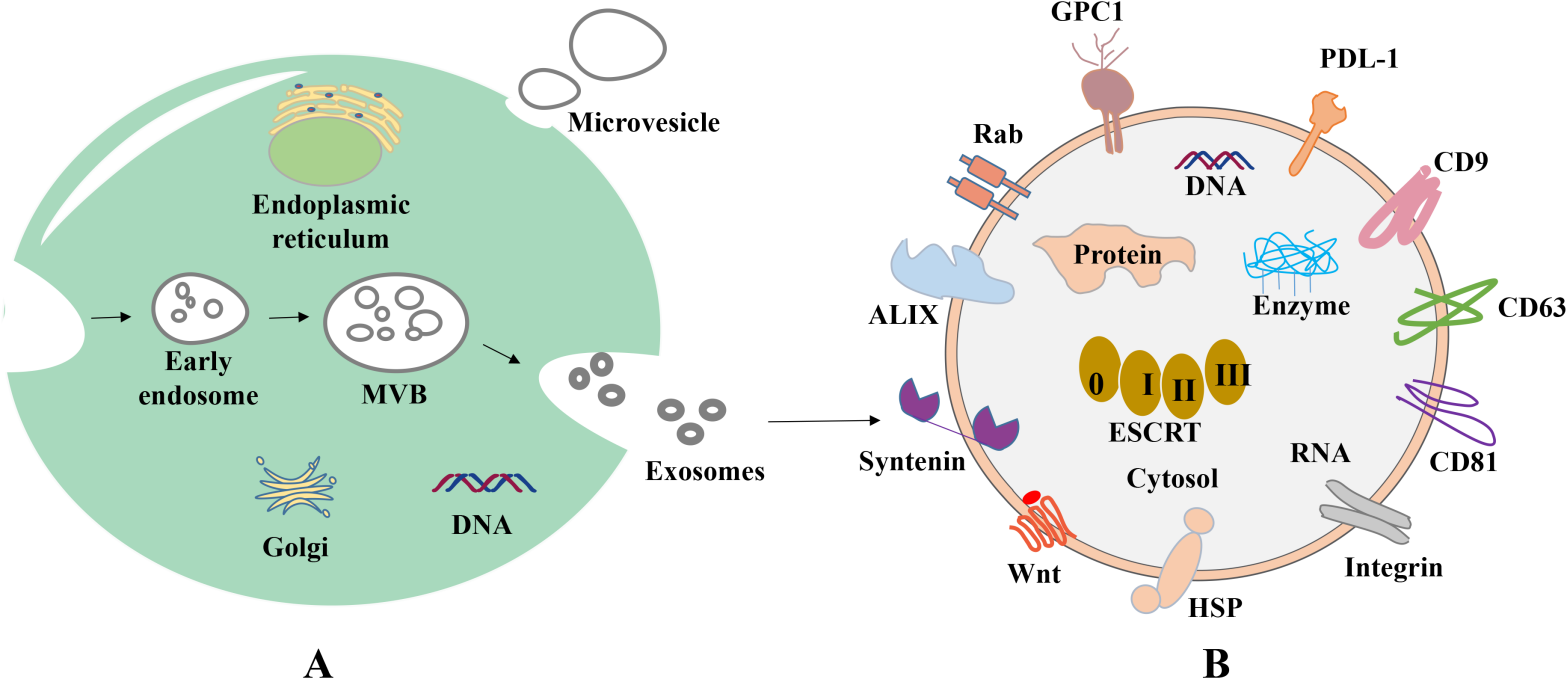
Biogenesis and composition of exosomes. **(A)** Origin of exosomes. First, plasma membrane invagination of donor cells forms early endosomes, which further mature into late endosomes. During the maturation, the membrane of early endosomes invaginates inwardly to form ILVs. Endosomes with ILVs are often referred to as MVBs. When MVBs fuse with the plasma membrane, ILVs are released into the extracellular space and termed as exosomes. **(B)** Common proteins of exosomes. Tetraspanin proteins (CD9, CD63, CD81), PD-L1, Integrins; Wnt protein, ALIX, Syntenin, HSPs, GPC1, Rabs, Flotillin, etc.

Extracellular vesicles (EVs) are a collective name that refers to nanoscale vesicles that are actively released by cells (24). Exosomes are formed through the fusion of multi-vesicular vesicles with the cell membrane, with a diameter of 50–200 nm, and microvesicles are formed through the direct outgrowth of the cell membrane, with a diameter of 200-2,000 nm. Moreover, apoptotic vesicles are formed through the shrinkage and fragmentation of apoptotic cells, with a diameter of 500-2,000 nm (25) (**Figure 1A**).

In 1983, exosomes were initially isolated from sheep reticulocytes. Through the investigation of the transferrin receptor during the maturation of reticulocytes, Johnston et al. discovered that the mechanism of the transferrin receptor is lost when exosomes are created during the maturation of erythrocytes (26). There are a variety of cell types that are capable of actively producing exosomes, including human umbilical vein endothelial cells, reticulocytes and immune cells, such as lymphocytes, macrophages, dendritic cells, natural killer cells, stem cells, endothelial cells and neuronal cells (27–30). Exosomes may be produced under both healthy and pathological conditions, and exosomal development includes initiation, endocytosis, the creation of multivesicular bodies and release. The creation of exosomes begins with the endocytosis of vesicles, leading to the development of early sorting endosomes via the invagination of the cell membrane. Subsequently, these endosomes evolve into late sorting endosomes (31), and these bud inward to form multivesicular bodies (MVBs). MVBs ultimately fuse with the plasma membrane and discharge their contents into the extracellular environment, and these are referred to as exosomes (**Figure 1A**). Moreover, released exosomes may be directed to other cells using a variety of cell surface proteins, including tetraspanning proteins (32–34).

**Figure 2:**
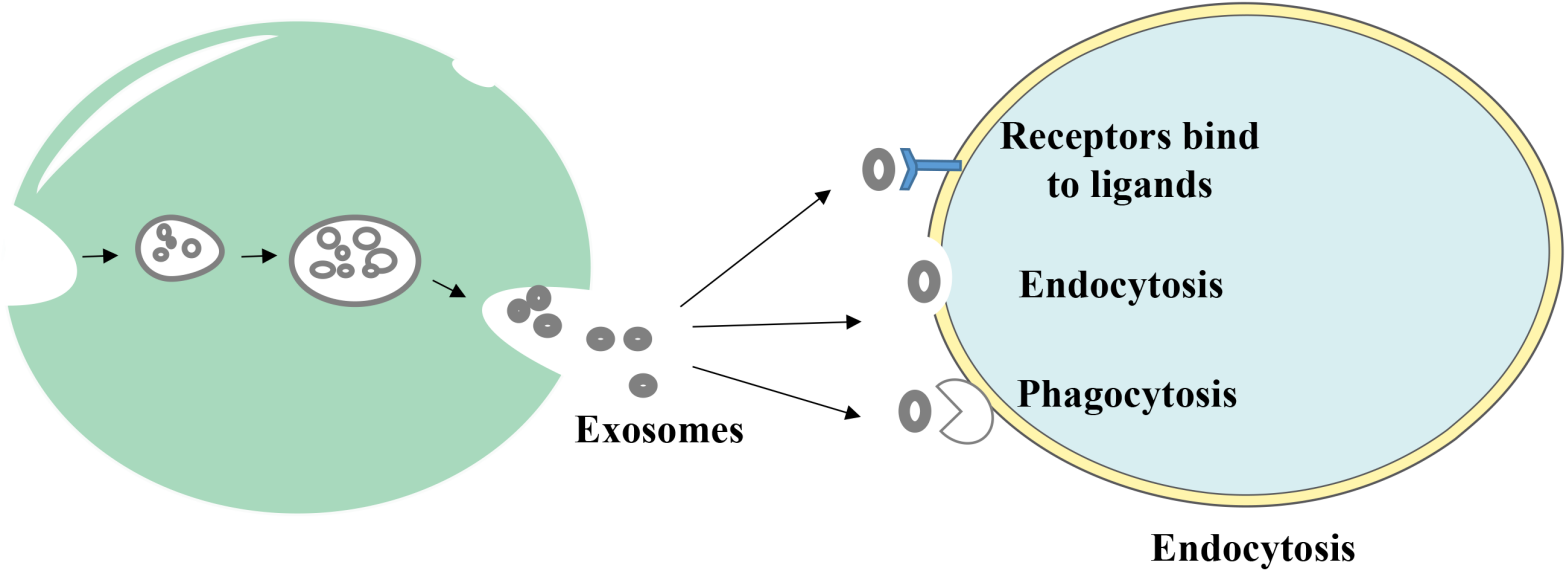
The way exosomes bind to receptor cells. Exosomes transmit information to receptor cells through three main pathways: (1) receptor-ligand interactions, (2) direct fusion with the cell membrane, and (3) endocytosis via phagocytosis.

Endosomal sorting complexes (ESCRT) are required for translocation-dependent processes. Significantly, ESCRT and non-ESCRT mechanisms (35) play a key role in the production of exosomes. ESCRT-0, ESCRT-I, ESCRT-II and ESCRT-III are the four functional subcomplexes involved in the ESCRT mechanism, and this is comprised of ~30 proteins. Particularly, the aforementioned subcomplexes are required for exosomal biogenesis. Lipids and associated proteins, such as the transmembrane tetraprotein CD63, play key roles in the ESCRT pathway, and these do not require any other proteins. Results of previous studies demonstrated that specific structures, such as lipid rafts and proteins with a four-transmembrane structural domain, may play a crucial role in the creation of certain exosomes (36, 37). Notably, exosomes transfer information to receptor cells via three primary routes (**Figure 2**); namely, i) Receptor-ligand interactions; ii) direct fusion with the cell membrane; and iii) endocytosis via phagocytosis.

## 3 Proteins of exosomes

At present, research is focused on exosomal proteins due to their unique biological functions and the key roles that they play in regulating the tumor microenvironment (TME). Some exosomal proteins are embedded on the surface of the membrane, and some are completely encased in the membrane (**Figure 1B**). Importantly, certain proteins, such as CD63, TSG101 and Alix, have been recognized as biomarkers for exosomes, while others, such as Calnexin, may function as negative markers for exosome identification (38). Specific proteins on exosomes, such as epidermal growth factor receptor (EGFR), Ephrin type-A receptor 2 (EphA2) and Epithelial cell adhesion molecule (EpCAM), are increasingly used to distinguish between tumor-derived exosomes and non-tumor-derived exosomes (39). Metastatic ovarian cancer cells release numerous exosomes carrying E-cadherin, which is an inducer of angiogenesis (40). Expression levels of exosomal lipopolysaccharide-binding protein and E-cadherin were used to identify non-small cell lung cancer (NSCLC) and ovarian cancer cells with metastatic phenotypes (41). Results of a previous study revealed that in patients with head and neck squamous cell carcinoma, exosomal Programmed cell death ligand 1 (PD-L1) expression levels were associated with disease progression, UICC stage and lymph node invasion (42). Moreover, the detection of PD-L1 positive exosomes in blood samples from patients with pancreatic ductal adenocarcinoma was associated with poorer survival (43). As a key immune checkpoint molecule, the expression levels of PD-L1 directly reflect the ability of tumor cells to evade immune surveillance. It has been found that revealed that PD-L1 inhibits T cell function and promotes immune escape through binding to PD-1 on the surface of T cells (44). In addition, epigenetic studies revealed that the demethylation status of the PD-L1 promoter is positively correlated with its expression levels (45). Collectively, these studies suggest that exosomal surface proteins may exhibit potential in the diagnosis and prognosis prediction of cancer; thus, exhibiting potential in monitoring treatment response. In addition, exosomal surface proteins may contribute to further understanding the mechanisms underlying exosome biogenesis (46–51), targeting (52, 53) and interaction (54–57). The main types of exosomal surface proteins and their functions are displayed in **Table 1**.

**Table 1:** Exosomal surface proteins and their roles.

Exosomal proteins	Roles	References
Major histocompatibility complex (MHC): MHC class I, MHC class II	Antigen presentation to induce an immune response	(53)
Tetraspanins (CD9, CD63, CD37, CD81, CD82, CD53)	Protein scaffolding and anchoring in cellular membranes. CD9, CD63, and CD81 are present at high levels in exosomes, are often used as exosome biomarkers, and can influence exosome biogenesis and composition	(47, 48)
GTPase, Annexins, Flotillin, Rab GTPases	Crucial in intracellular vesicle transport, including endosome recycling and MVB trafficking to lysosomes. They can mediate intraluminal vesicle budding and tethering of MVBs to the plasma membrane	(49–51)
Glycoproteins (β-galactosidase, O-linked glycans, N-linked glycans)	Specifically interact with receptors and enable the specificity of exosome targeting	(53, 54)
Fas ligand, TNF receptor, Transferrin receptor	Exosome targeting and signaling, including the induction of apoptosis and iron transport	(55, 56)
Integrin-α, integrin-β, P-selectin	Mediate the interaction, attachment, and membrane fusion with the target cell	(53)

## 4 Function of exosomes in cancer

Research has focused on the role of exosomes in tumors due to the key role they play in intercellular communication. Exosomes that originate from tumor cells or stromal cells are associated with all phases of cancer development. These stages include tumor growth and cell proliferation, the prevention of cell death, angiogenesis, immune evasion, invasion and metastasis. It has been shown that exosomes may play a key role in the intricate biological interactions that take place between tumor cells and the TME (58). Interestingly, there are numerous different physiologically active chemicals that are carried by exosomes. These compounds are critical signals for reprogramming the TME for the induction of carcinogenesis (59). For example, delta-like 4 protein (DLL4) plays a key role in the promotion of cancer-associated TME alterations (60) and the expansion of vascular branching. The presence of DLL4 in individuals with colorectal cancer (CRC) may be associated with aggressiveness of the tumor and negative clinical outcomes (61). Moreover, tumor cell-derived exosomes may function as catalysts for epithelial-to-mesenchymal transition (EMT) and tumor metastasis. This is accomplished by stimulating quiescent cancer cells to actively metastasize via the use of a wide range of signaling molecules, such as Notch1 and HIF1α (62, 63). It has been shown that tumor-derived exosomes may assist tumor cells in evading monitoring of the immune system, developing resistance to chemotherapy and further promoting the growth of the tumor. Results of previous studies revealed that tumor-derived exosomes decrease the cytotoxicity of natural killer cells and cytotoxic T-lymphocytes. Particularly, these cells are essential components of the immune response, which plays a key role in preventing the growth of tumors (64, 65). Previous research has shown that tumor-derived exosomes may promote the polarization of immature macrophages into M2 macrophages and exhibit anti-inflammatory activity, both of which are beneficial to the continued spread of the tumor. Interestingly, angiogenesis is a fundamental physiological process during carcinogenesis that involves numerous steps. A wide range of angiogenic factors, including vascular endothelial growth factor, interleukin, transforming growth factor-β and fibroblast growth factor, are located inside exosomes (66). These factors play a crucial role in promoting the proliferation and migration of endothelial cells, and are essential in the generation of tumor angiogenesis (67, 68).

## 5 Potential use of exosomal proteins in the diagnosis of cancer

**Figure 3:**
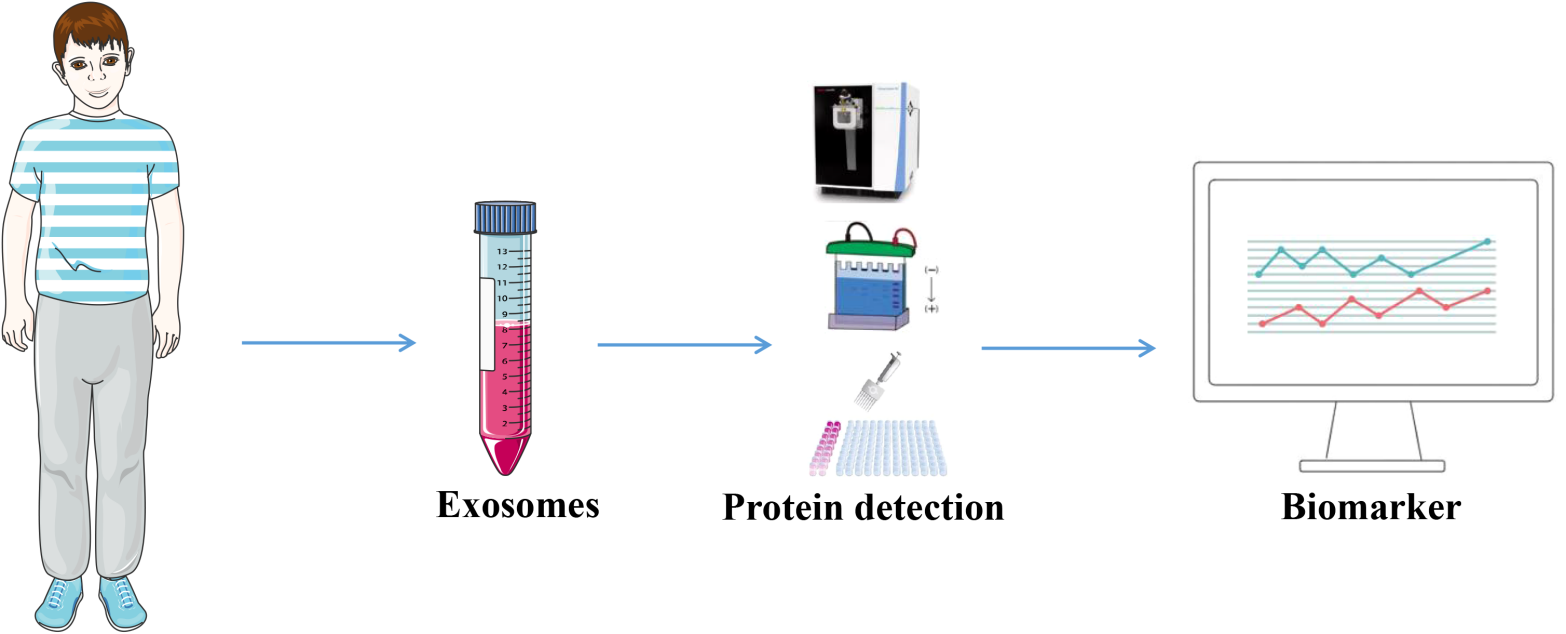
Tumor-derived exosomal proteins were used as diagnostic markers. Exosomes were extracted from body fluids from tumor patients and analyzed by Western Blotting, ELISA, proteomics and other exosomal proteins for diagnosis.

Given their disease-specific alterations, exosomal proteins have emerged as potential biomarkers for cancer. Exosomes that are produced from tumors carry a large amount of data regarding the biology of cancer cells (69). Levels of exosomal proteins in patients with cancer are considerably higher than those observed in healthy individuals. As a result, the identification of proteins in exosomes is sensitive and advantageous for the early diagnosis of cancer. Thus, the identification of exosomal proteins may exhibit potential in the diagnosis of cancer and in predicting patient prognosis (70). The tumor-derived exosomal protein analysis process is displayed in **Figure 3**, and **Table 2** summarizes the exosomal proteins that may exhibit potential as diagnostic markers in various tumors.

**Table 2:** Exosomal proteins as potential diagnostic markers in various tumors.

Type of cancer	Protein marker	Sample type	Isolation technique	Diagnostic accuracy	Reference
Colorectal cancer	Glypican-1	Plasma	Immunocapture assays	N/A	(72)
	CD147	Serum	Immunocapture assays	AUC 0.82	(73)
Gastric cancer	GKN1	Serum	Ultracentrifugation	Sensitivity 91.2%, specificity 96.0%, AUC 0.94	(79)
	HER2	Serum	precipitation	Sensitivity 66.7%, specificity 74.2%, AUC 0.74	(83)
Pancreatic cancer	Glypican-1	Serum	Ultracentrifugation	Sensitivity 100%, specificity 80%, AUC 1.0	(87)
	EGFR and HER2	Serum	Ultracentrifugation	Sensitivity 85%	(88)
Prostate cancer	Transmembrane protein 256	Urine	Ultracentrifugation	Sensitivity 94%	(91)
Bladder cancer	56 proteins	Urine	Ultracentrifugation	N/A	(93)
Cvarian cancer	Claudin-4	Plasma	Ultracentrifugation	Sensitivity 98%, specificity 51%	(97)
Non-small cell lung cancer	LRG1	Urine	Ultracentrifugation	N/A	(101)
	IGHV4-4 and IGLV1-40	Plasma	Ultracentrifugation	AUC0.95	(104)
	Versican	Plasma	Test kit	Sensitivity 65.1%, specificity 83.2%, AUC 0.81	(105)
Breast cancer	Fibronectin	Plasma	Immunoaffinity capture	Sensitivity 85.4%, specificity 61.8%, AUC 0.79	(105)
Melanoma	Caveolin-1	Plasma	Ultracentrifugation	Sensitivity 68%	(110)
Parkinson disease	LRRK2	Urine	Ultracentrifugation	AUC 0.84	(112)

N/A, Not applicable; AUC, Area Under Curve.

### 5.1 Digestive cancers, including colorectal cancer, stomach, liver and pancreatic cancer

Exosomes include a variety of proteins that may exhibit potential as diagnostic markers for CRC. These proteins include carcinoembryonic antigen (CEA), epidermal growth factor receptor, mitogen-activated protein kinase and keratin (71). Previous studies have found that these proteins may be used for the diagnosis of CRC. In the previous study, Glypican-1^+^ (GPC1^+^) exosomes were successfully separated from the tissues and plasma of CRC. Compared with controls, the expression of GPC1^+^ exosomes were markedly higher in the tumor tissues and plasma of patients with CRC prior to surgical treatment (72). CD147-positive exosomes have been shown to be effective as a diagnostic indicator for colorectal cancer, with an AUC of 0.827 (73). In addition, exosomes produced from CRC cells have been subjected to proteomic analysis, which revealed the unique expression of a number of metastatic factors. Importantly, these factors include hepatocyte growth factor receptor, S100A8, S100A9 and tenascin C (74, 75). A histological examination of phosphorylated proteins in exosomes obtained from metastatic CRC cells revealed that the levels of phosphorylated proteins in these exosomes were higher than in those located in exosomes derived from non-metastatic CRC cells (76, 77). Further investigations are required to determine the potential use of exosomal proteins in the detection and treatment of CRC.

According to the World Cancer Report, gastric cancer (GC) is the fourth most common cancer worldwide, and the second highest cause of cancer-associated death (78). Yoon et al. revealed that the blood GKN1 levels of healthy controls were markedly higher than those of patients with GC. Especially, the sensitivity and specificity of GC were 91.2 and 96.0%, respectively (79). In addition, results of a previous study revealed that serum GKN1 levels may aid in distinguishing individuals with CRC from those with CRC, liver, lung, breast, pancreatic, ovarian or prostate cancers that exhibited area under the curve (AUC) values of >0.94 (80). Interestingly, exosomes produced from GC cells possess the ability to promote activation of the NF-ÙB pathway in macrophages, leading to the advancement of cancer (81). It has been found that the involvement of exosomes carrying tetraspanin 8 may be associated with the proliferation and invasion of cells in GC, and that tetraspanin 8 is an independent factor in determining the prognosis of patients with GC (82). Serum-derived exosomes of HER2 as a promising biomarker for advanced gastric cancer had an area under the ROC curve of 0.746, a sensitivity of 66.7% and a specificity of 74.2% (83). Collectively, these results imply that exosomal proteins may exhibit potential as diagnostic and prognostic indicators for GC.

When liver cancer is in its early stages, the symptoms that patients experience are often non-specific, and late diagnosis leads to limited treatment options. Protein levels in serum exosomes obtained from patients with hepatocellular carcinoma (HCC) and a healthy cohort were analyzed by Arbelaiz et al, who demonstrated that the expression levels of G3BP and PIGR were significantly elevated in patients with HCC. Moreover, exosomal proteins were more effective than AFP in predicting HCC (84). Fu et al. revealed that the amount of SMAD3-positive exosomes generated from HCC cells was positively correlated with the staging and pathologic grading of HCC; however, this was negatively correlated with the disease-free survival of patients with HCC following surgery (85). In addition, 14-3–3 protein expression levels were associated with a larger tumor size, poorer tumor differentiation and more advanced TNM staging. Wang et al. revealed that the amount of exosomal 14-3–3 proteins were elevated in HCC cell sources. Notably, 14-3–3 protein expression levels were also associated with increased tumor size (86).

In addition, exosomal proteins may exhibit potential in the pathological detection of pancreatic cancer (PC). Buscail et al. revealed that exosomes produced from PC cells that were positive for GPC1 exhibited a high level of accuracy, with an AUC of 1.0, a sensitivity of 100% and a specificity of 80% (87). Combined detection of the exosomal membrane proteins EGFR and HER2 improves the diagnostic sensitivity of pancreatic cancer (85%) and is particularly effective in CA19-9-negative patients (88). Individuals with metastatic pancreatic cancer exhibited markedly increased levels of macrophage migration inhibitory factors (MIFs), compared with those who did not experience progression of PC. These findings suggested that exosomal MIFs may exhibit potential as predictive markers for liver metastasis (89). Thus, further investigations are required to determine the specific function of exosomal proteins in PC.

### 5.2 Urinary cancers, including prostate, bladder and ovarian cancer

Plasma prostate-specific antigen, also referred to as prostate specific antigen (PSA), is a biomarker that is frequently used for the purpose of detecting and monitoring prostate cancer. Nilsson et al. revealed that urine exosomes derived from individuals with prostate cancer exhibited high expression levels of β-catenin, prostate cancer gene-3 (PCA-3) and several other markers associated with prostate cancer. These findings highlight the potential for urine exosomes in identifying and monitoring cancer (90). Moreover, Øverbye et al. conducted a study on urine exosomal proteins in a group consisting of 16 individuals diagnosed with prostate cancer and 15 healthy controls. Results of this previous study revealed that 246 proteins were differently expressed in both groups of patients. Out of 17 of these proteins, all 17 exhibited a sensitivity of >60% and a specificity of 100%, with TM256 exhibiting the highest level of sensitivity at 94% (91). Proteomes of urine exosomes obtained from patients with prostate cancer and healthy participants were compared, and the results revealed that TM256 and ADIRF exhibited the greatest diagnostic value (91). Collectively, these results revealed that urine exosomal proteins may exhibit potential as a source of enrichment for prostate cancer indicators.

The tumor-associated calcium signaling 2 (TACSTD2) protein, which exhibits potential in the detection of bladder cancer (92), is one of the 29 urine exosomal proteins that have emerged as novel prospective biomarkers. Lee et al. carried out proteome characterization of urine-derived exosomes from ten healthy controls and ten patients with bladder cancer. Results of this previous study revealed that 56 proteins were highly expressed in the urinary exosomes of patients with bladder cancer (93). In addition, the expression levels of CD36 and CD44 in exosomes were identified via immunoblotting and flow cytometry, and results of this previous study revealed substantial differences in the expression of CD36 and CD44 between healthy individuals and patients with bladder cancer (94).

It is estimated that >70% of ovarian cancer diagnoses are made at an advanced stage (95), leading to high levels of mortality in female patients. Research focused on epithelial cell adhesion molecules and CD24 in exosomes produced from ovarian cancer has led to a novel option for the early diagnosis of ovarian cancer (96). The study shows that serum-derived exosome Claudin 4 steadily increased with the advancement of cancer in patients with ovarian cancer (97). In addition, expression levels of the exosome surface marker, HSP70, were higher in exosomes formed from OC cells, compared with those obtained from healthy controls. Moreover, study find that the serum of individuals with OC exhibited a significant quantity of exosomes that expressed HER2. Thus, further investigations are required to determine and distinguish exosomal populations, and investigate the biological activities of exosomal proteins in biological organisms (98).

### 5.3 Thoracic cancers, including lung and breast cancer

Exosomal proteins may exhibit potential in the diagnosis of non-small cell lung cancer (NSCLC). Significantly, 2% of exosomes were located in chronic inflammatory lung tissue (99). Wu et al. revealed that 80% of exosomes recovered from NSCLC biopsies were positive for EGFR. Interestingly, EGFR, K-Ras, claudin1, claudin3 and RAB family proteins were among the potential diagnostic indicators discovered by Park et al (100), when exosomes were recovered from pleural effusions of patients with NSCLC. Results of proteomic mass spectrometry (101) revealed that human leucine-rich alpha-2-glycoprotein 1 (LRG1) was concentrated in urine exosomes, and was expressed at higher levels in patients with NSCLC, compared with healthy individuals. Sandfeld et al. used 49 antibodies to identify EV proteins obtained from 431 patients with lung cancer and 150 healthy individuals (102). Moreover, a diagnostic model containing 30 exosomal proteins was developed in a further study, with a sensitivity and specificity of ~75% (103). The model was constructed using an exosome array to harvest exosomes from the blood of patients with NSCLC. *Yang* et al. identified plasma-derived exosomal immunoglobulins IGHV4–4 and IGLV1–40 as new non-small cell lung cancer biomarkers (104). A recent study has identified plasma exosomal versican as a potential diagnostic marker for non-small cell lung cancer (105). Thus, exosomes and associated components may exhibit potential in the early identification of lung cancer.

At present, breast cancer is considered the most prevalent form of cancer among females. In patients with metastatic breast cancer, the 5-year survival rate is ~20%, despite >50% of patients with breast cancer developing metastases following systemic intervention. Melo et al. demonstrated that 75% of patients with breast cancer exhibited greater levels of exosomal GPC1 expression, compared with healthy controls (106). The diagnostic value of fibronectin and developmental endothelial locus-1 in exosomes produced from breast cancer cells was reported by Moon et al, with an AUC of 0.961, a sensitivity of 94.70%, and a specificity of 86.36% (107, 108). Moreover, the unique expression pattern of exosomal survivin-2B in serum may exhibit potential as an indicator for breast cancer in its early stages (109).

### 5.4 Other cancers

In a wide range of malignancies, exosomal proteins have been proposed as potential diagnostic and prognostic indicators. The study noted that numerous clinical samples may include a high number of exosomal proteins that are specific to melanoma (110), such as caveolin-1. Serum exosomes were analyzed in patients with glioblastoma, and results of a previous study revealed that EGFR, EGFRvIII and CD63 were expressed at high levels (111). Fraser et al. discovered that leucine-rich repeat kinase 2 (LRRK2), which is abundant in urine exosomes, may exhibit potential as a biomarker for Parkinson’s disease (112). In addition, the existence of spongioblastoma-specific EGFR variant type III (EGFRV III) was discovered through the detection of serum exosomes from 25 individuals with spongioblastoma (113). *Reale* recently showed that proteomic characterization of human plasma extracellular vesicles provides important implications for the development of multiple myeloma diagnostics (114). A recent study identified 199 common proteins in exomes secreted from Synovial sarcoma cells, with the monocarboxylate transporter 1 (MCT1) as a novel surface marker, highly expressed in SS patient-derived exosomes compared with healthy individuals (115). Wang et al. found that DIO3OS could be a potential biomarker for thyroid-like cancer (116). With further research, exosomal proteins are increasingly used in various cancer diagnostics.

## 6 Potential therapeutic value of exosomal proteins in cancer

Exosomal proteins not only exhibit potential as a molecular marker for the diagnosis of cancer, but also demonstrate therapeutic potential due to their unique biological characteristics. At present, research is focused on use of engineered exosomes for the targeted distribution of anti-cancer therapy. Results of a previous study revealed that modified exosomes that include adriamycin may be more effective in targeting tumors and inhibiting their growth (117). An additional investigation was conducted in which the parental cells of exosomes were modified to produce Lamp2b linked to αv integrin-specific iRGD peptide. Importantly, this peptide demonstrated sufficient tumor-targeting characteristics in prostate, breast and cervical cancer models (118). In addition, The study noted that a subtype of MVBs, known as inhibitory protein structural domain 1-mediated microvesicles, aids in the transportation of NOTCH receptors to target cells and the stimulation of downstream gene expression (119). Votteler et al. discovered an innovative method for hybridizing exosomes using envelope protein nanocages, also known as EPNs. Through the process of membrane attachment and self-assembly, extracellular polymeric nanoparticles (EPNs) transfer their payload into the cytoplasm of recipient cells (120). This is accomplished through binding a variety of designed proteins.

In addition, the immunological activity of exosomes may allow them to be used as drug transporters in cancer immunotherapy or as vaccines (121). As exosomes are responsible for transporting a large number of tumor antigens, they may play a role in antigen presentation, exhibiting potential as cancer vaccines (122). Moreover, Hsp70 plays a key role in activating dendritic cells and monocytes, which in turn stimulates immunological responses that are mediated by tumor-derived exosomes (123). Hsp70 also plays a key role in promoting the release of granzyme B from natural killer cells, ultimately leading to the induction of apoptosis in tumor cells (124). Interestingly, HSP70 may function as an exosome surface antigen, eliciting anti-tumor antibody responses. Thus, exosomal proteins exhibit potential in the treatment of a wide range of malignancies. Clinical trials of exosomes in cancer therapy have focused on the following areas, with specific applications including as drug delivery vehicles, immunomodulators, diagnostic markers, and combination therapy tools. Most of the trials are currently in the early stages (phase I/II), but the multifunctional properties of exosomes make them have great potential in personalized cancer therapy (**Table 3**). With advances in bioengineering technology, more precise clinical applications may be realized in the future.

**Table 3:** Clinical trial applications of exosomes in cancer therapy.

Type of cancer	Mechanisms	Clinical trial phase	NCT Number
Pancreatic	Carrying chemotherapeutic agents/nucleic acids to target tumors	Phase I/II	NCT03608631
Non-small cell lung cancer	Exosome vaccine or immunomodulation	Phase I/II	NCT01159288
Colid tumor	Enhancing the efficacy of radiotherapy/immunotherapy	Phase I/II	NCT05375604
Colorectal cancer	Anti-tumor cytotoxicity T lymphocyte response	Phase I	undisclosed
Triple-negative breast cancer	Inhibits tumor growth and enhances sensitivity to radiotherapy	Phase I/II	NCT04491240
Liver cancer	Delivery of Sorafenib	Phase I/II	NCT04868344
Gastric cancer	Activation of T-cell immunity by dendritic cell exosomes	Phase I/II	NCT04313647

## 7 Discussion

As a novel form of liquid biopsy markers, exosomal proteins may demonstrate potential in the diagnosis of cancer. In recent years, tumor-derived exosomal proteins have demonstrated unique diagnostic value, and key advantages of these proteins include the following factors: i) Disease-specific enrichment, where the protein composition of exosomes highly reflects the physiological and pathological status of their parent cells, particularly tumor cell-derived exosomes (TEXs). These exosomes carry a large number of tumor-related proteins, such as EGFR, HER2, PD-L1, MET and GPC1, which may be used as cancer-specific markers; ii) a high level of stability, making them suitable for long-term storage and the detection of clinical samples. The lipid bilayer membrane structure of exosomes protects the internal proteins from proteases in the blood. Notably, this is more stable than free proteins or ctDNA due to the strong anti-degradation ability; iii) the potential clinical application of minimally invasive/non-invasive testing. Especially, small volumes of bodily fluid, such as 1 ml of blood or urine, is required for routine tests, mitigating limitations associated with tissue biopsy and sampling; and iv) multi-dimensional diagnostic information integration. Multiple protein marker combinations may be analyzed at the same time to improve diagnostic accuracy.

Although exosomal proteins exhibit potential in the diagnosis of cancer, limitations may remain, leading to limited use in clinical practice. Limitations may include the following factors: i) Complexities in the standardization of isolation and purification techniques. At present, exosome isolation requires numerous techniques, such as ultracentrifugation, size-exclusion chromatography, polymer precipitation and immunoaffinity capture. However, these methods exhibit notable differences in recovery rate, purity and integrity of exosomes. Contaminants, such as lipoproteins and protein aggregates in blood samples, often co-precipitate with exosomes, affecting downstream analysis. Although the International Society for Extracellular Vesicles (ISEV) has proposed the MISEV guideline, a globally recognized standardized process is yet to be developed, resulting in poor comparability of data; ii) analytical challenges associated with tumor heterogeneity. Importantly, exosomes in bodily fluids may be derived from tumor cells, immune cells and platelets. Tumor-derived exosomes may account for >1% of total exosomes, and therefore require enrichment with highly specific markers. In addition, the composition of exosomal proteins changes continuously with tumor progression and treatment response, and a dynamic monitoring system is required; and iii) complexities in large-scale application and the translation to clinical practice. The majority of studies include small-scale cohorts and lack validation through multi-center, prospective clinical trials. High-precision separation and detection technologies, such as nanoflow or single exosome analysis are costly, and there may be barriers associated with their use in clinical practice.

Exosomal proteins exhibit high levels of potential in the diagnosis of cancer; however, interdisciplinary collaboration, optimization of technical processes and the establishment of a globally uniform standardization system are required for their use in clinical practice. Future research should focus on developing high-purity and high-throughput separation technologies, such as microfluidic chips and aptamer capture. In addition, further investigations focused on the establishment of a multi-omics integrated analysis process are required, to integrate proteins, RNA and metabolites. Future research should also focus on the use of AI-assisted marker screening to improve the efficiency of data analysis. The aforementioned investigations may lead to evolution of the field of liquid biopsy, revolutionizing the early diagnosis and treatment of cancer.

As emerging tumor markers and therapeutic carriers, exosomal proteins have demonstrated revolutionary potential in the diagnosis and treatment of cancer. In the diagnosis of cancer, the unique molecular features and high stability of exosomes may provide a novel basis for early screening, precise typing and the dynamic monitoring of tumors. As a natural delivery system, exosomal proteins may exhibit potential in the treatment of cancer, leading to the development of targeted therapies and immunomodulation. However, future investigations are required to overcome key challenges, including isolation standardization, up-scaling of production and clinical validation. With the integration and development of interdisciplinary technologies, such as nanotechnology, multi-omics analysis and artificial intelligence, exosomal proteins may lead to considerable developments in the diagnosis and treatment of cancer, aiding the development of precise and personalized medicines. Notably, future investigations should focus on establishing a standardized technical system through multi-center clinical trials, and exploring the specific molecular mechanisms associated with exosomes.
